# Training in Poor-Law Infirmaries

**Published:** 1907-08-24

**Authors:** 


### Nursing Administration.

### Training in Poor-Law Infirmaries.

### I.—Its Drawbacks and Triumphs.

The superintendence of nursing in Poor-law infirmaries is carried on under conditions which differentiate them in many important particulars from hospital work. The question as to whether good nurses can be trained under the system has been settled once and for all. A stream of highly trained and competent nurses has now begun to issue from the best Poor-law training schools, and so inadequate is the supply of well-trained nurses for the demand that the public would infallibly feel the pinch of necessity should this source of supply for any reason be checked. The more closely the Poor-law Infirmary can succeed in following the lines of the best hospital training for its nurses, the better prospect infirmary-trained nurses will have on completing their course, and as a natural consequence the better will be the class of women offering themselves for training in these establishments. The differences in organisation between the voluntary hospital and the rate-supported institution are partly inherent to these distinct branches of the public service, and partly accidental. Perfection in the training of nurses has not yet been attained in either class of institution, but it is generally recognised that the hospital has so far made greater progress in that direction than the infirmary. Hence, it is very important to consider in what particulars the Poor-law training is capable of expansion, and to what extent its limitations must be borne with as fixed, however they may be compensated by extra privileges.

The following points will occur to even a superficial observer as distinguishing the Poor-law from the voluntary hospital training school:—

1. The relatively small size of the staff compared with the number of beds.
2. The absence in most infirmaries of any extra staff to take special cases or relieve the regular staff for leave or holidays.
3. The small proportion of medical attention, two doctors commonly sufficing for institutions containing several hundred beds.
4. The large proportion of chronic cases.
5. Security of tenure in appointments leading to the practically immutable position of the sisters.
6. The absence of any power of dismissal on the part of the matron, even with obviously unsuitable probationers.
7. The total want of public interest displayed towards the institution.
8. Government by Guardians, the majority of whom have no sympathy with infirmary work, instead of by keenly interested governors, who are themselves subscribers.
9. An admirable system of superannuation and pension, putting a premium upon long service.
10. Complete absence of all financial strain.

All these points tell more or less in the training of probationers. The first four are commonly considered the most conspicuous features of the infirmary, but perhaps they affect the character of the training less than the administrative details we have enumerated under the later headings. They alter the nature of the duties to be carried out, it is true, but then it has been shown over and over again that efficient nurses can be evolved from the most varied types of institution provided only that due care is taken to keep the balance of training even. Now this quality of evenly balanced training and experience is exactly what is achieved in the Poor-law system. The nurses may not see a great variety of work. Big surgical operations are comparatively rare and advanced obstetrical work, or obscure medical diseases may be seldom encountered. But such as the work is, and its character is more generally representative than is often understood by purely hospital trained nurses, the infirmary nurse gets her fair share of it all. She is not kept to one branch of nursing until she almost forgets there is any other. The large wards where medical and surgical cases are received without classification are each a hospital in themselves. The probationer is not confined to mere routine attendance on others. She is not cut off altogether from any practice in performing important nursing processes, because there are too many probationers in training to admit of all getting a fair chance. It is rather the other way. She is compelled to be self reliant by virtue of the very defects in organisation. If there is no holiday staff it follows that juniors get opportunities of showing their mettle. If there is little help to be looked for from the overworked medical officers there is all the more practice in dressings and bandaging, all the more need for precision and conciseness in preparing for the rapid visit. If there is less excitement in the interchange of patients, less interest in the presentment of disease, when many are incurable, there ought surely to be more human interest than is possible in the hurried atmosphere of the general hospital, where patients succeed one another too rapidly to leave much impression on the staff. And, lastly, though there may be less direct inspiration from medical zeal than is reflected upon nurses in general hospitals, there is often far more personal attention bestowed by the medical officers on details promoting the training of the nurses than is the case in hospital. The excellent results shown in tests by outside examiners are a proof that these advantages are having full effect on the standard of training. The Poor-law infirmaries do not brand their own herrings.

The more serious drawbacks to infirmary nursing are gradually disappearing. Year by year the proportion of nurses to beds is decreasing, as the Guardians come to perceive the importance of securing a good class of nurse for infirmary work. If the position of the probationer in the smaller infirmaries and in the sick wards of workhouses is still a painful anomaly, it may be fairly claimed for the large London and Provincial infirmaries that the battle as regards training has been won.

